# Incidence, Predictors, and Outcome of Paravalvular Leak after Transcatheter Aortic Valve Implantation

**DOI:** 10.1155/2020/8249497

**Published:** 2020-05-22

**Authors:** Abdullah Hagar, Yijian Li, Xin Wei, Yong Peng, Yuanning Xu, Yuanweixiang Ou, Zijie Wang, Xi Wang, Jageshwar-Prasad Shah, Vivendar Sihag, Mao Chen, Yuan Feng

**Affiliations:** ^1^Department of Cardiology, West China Hospital, Sichuan University, 37 Guoxue Alley, Chengdu 610041, Sichuan, China; ^2^Cardiology Department, B & B Hospital, Gwarko, Lalitpur, Nepal

## Abstract

**Background:**

Paravalvular leak (PVL) is common after transcatheter aortic valve implantation (TAVI) and has been linked with worse survival. This study aimed to investigate the determinants and outcome of PVL after TAVI and determine the role of aortic valve calcification (AVC) distribution in predicting PVL.

**Methods and Results:**

This was a retrospective cohort study of 270 consecutive patients who underwent TAVI. Determinants and outcomes of ≥mild PVL were assessed. Matching rates of PVL jet with AVC distribution were calculated. AVC volume, larger annulus dimensions, and transvalvular peak velocity were risk factors for ≥mild PVL after TAVI. AVC volume was an independent predictor of ≥mild PVL. On the other hand, annulus ellipticity, left ventricular outflow tract nontubularity, and diameter-derived prosthesis mismatch were not found to predict PVL after TAVI. PVL jet matched, in varying proportions, with calcification at all aortic root regions, and the highest matching rate was with calcifications at body of leaflets. Moreover, matching rates were less with commissure compared to cusp calcifications. Mild or greater PVL was not associated with all-cause and cardiovascular mortality up to 1-year follow-up.

**Conclusion:**

≥mild PVL after TAVI is common and can be predicted by aortic root calcification volume, larger annulus dimensions, and pre-TAVI transvalvular peak velocity, with calcification volume being an independent predictor for PVL. However, annulus ellipticity, left ventricular outflow tract nontubularity, and diameter-derived prosthesis mismatch had no role in predicting PVL. Importantly, body of leaflet calcifications (versus annulus and tip of leaflet) and cusp calcifications (versus commissure calcification) are more important in predicting PVL. No association between ≥mild PVL and increased risk of all-cause and cardiovascular mortality at 1-year follow-up.

## 1. Introduction

Transcatheter aortic valve implantation (TAVI) is a well-established first-line therapy for severe symptomatic aortic stenosis (AS) patients who are at intermediate or higher surgical risk [1, 2]. Paravalvular leak (PVL) is common after TAVI and has been linked with worse survival [3]. Preprocedural multislice computed tomography (MSCT) is considered the most reliable method for measuring aortic root parameters in patients undergoing TAVI and has shown to be more advantageous in decreasing rates of PVL compared to echocardiography and, hence, has become the preferred imaging method for TAVI patients [4]. Some risk factors for developing PVL after TAVI have been identified [3, 5–8]. However, there is currently no integrated method which includes all parameters that may predict PVL after TAVI. We sought to conduct the present study to investigate the determinants and outcome of PVL after TAVI and to evaluate the role of aortic valve calcification (AVC) distribution in predicting PVL.

## 2. Materials and Methods

### 2.1. Patient Population

Data from 270 consecutive patients with severe symptomatic AS who underwent TAVI at west China hospital of Sichuan University, Sichuan, China, from April 2012 to November 2017 were retrospectively analyzed. Of these, 3 patients had preexisting surgical valve and 11 patients had no prostheses implantation due to potential risk of coronary occlusion or annulus rupture found during the procedure of predilatation. Thus, 256 patients were finally included. All included patients have undergone MSCT and transesophageal or transthoracic echocardiography (TEE, TTE) before TAVI for prosthesis sizing and selection of vascular access and TEE or TTE during the procedure for PVL assessment. The baseline surgical operative risk was calculated using the Surgeons Risk Score for Prediction of Mortality (STS score) [9].

Based on the severity of PVL after TAVI, patients were divided into two groups: ≥mild PVL group or <mild PVL group. In patients with ≥mild PVL, AVC distribution and PVL jet location were analyzed. Then we calculated the matching rates of AVC distribution and PVL jet for each aortic root region (annulus, body of leaflet, and tip of leaflet) first for all patients and then for each tricuspid aortic valve (TAV), bicuspid aortic valve (BAV) type I, and BAV type 0 subgroups. Finally, matching rates of cusps calcifications and commissures calcifications with PVL jet were analyzed. The study was approved by the institutional review board, and all patients provided written signed consent.

### 2.2. MSCT Acquisition and Image Analysis

**Figure 1:**
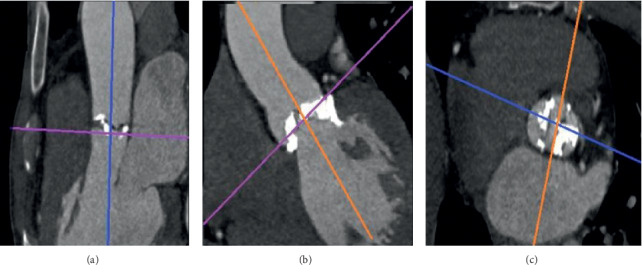
Multiplanar reconstruction used for the assessment of aortic root. (a) Single oblique sagittal view; (b) coronal view; (c) double oblique transverse view.

**Figure 2:**
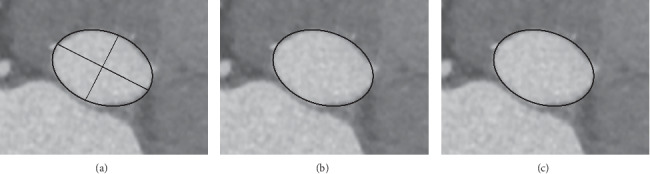
Aortic annular measurements on the MSCT. (a) Maximum and minimum annular diameters; (b) annular area; (c) annular circumference.

CT scans were performed using a 64-MSCT scanner (SOMATOM Definition Flash; Siemens Healthineers, Erlangen, Germany). Aortic root measurements were accomplished by analyzing pre-TAVI MSCT with OsiriX (OsiriX Foundation, Geneva, Switzerland) (Figure 1). The aortic valve annulus was defined as a plane including the lowest basal attachment points of the aortic valve leaflets in the left ventricular outflow tract (LVOT). MSCT measurements included minimum and maximum annular diameters, area, and circumference, as well as LVOT area. The mean annular diameter was calculated by taking the average of the minimum and maximum diameters. Measurements were performed using midsystolic MSCT images (Figure 2). The area of a completely expanded transcatheter heart valve (THV) was calculated by the following formula: (3.14 × radius^2^ measured in mm^2^). On this basis, prosthesis mismatch was calculated using the method described by Buzzatti et al. [10] as follows: ([mean diameter of the prosthesis/mean annulus diameter] × 100). Annular cover index was calculated as follows: ([THV area × area of the annulus/THV area] × 100). Annular ellipticity was calculated as ([maximum annular diameter − minimum annular diameter/maximum annular diameter] × 100) and LVOT nontubularity as ([annular area − LVOT area/annular area] × 100) using a method introduced by Condado et al. [11].

### 2.3. Analysis of Aortic Valve Calcification

**Figure 3:**
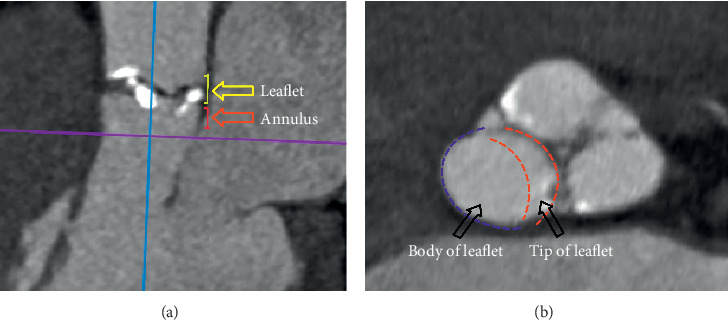
Anatomical regions of the aortic root. (a) Regions of the aortic valve in the craniocaudal axis along the long axis of the aortic valve/LVOT: annulus (from 3 mm above to 2 mm below the annular plane) and leaflet (from 3 mm above the annular plane to the superior edge of leaflets); (b) parts of aortic valve leaflet.

The analysis of calcification was performed using diastolic MSCT images at 75% of the RR interval using calcium volume scoring system [5, 12]. An adjusted threshold of 550 Hounsfield units was used for calcification quantification for most patients [5, 7]. Calcium quantification was performed by a cardiologist experienced in cardiovascular imaging. The aortic root was divided into the following regions in the craniocaudal axis along the long axis of the aortic valve/LVOT: annulus (from 3 mm above to 2 mm below the annular plane) and leaflet (from 3 mm above the annular plane to the superior edge of leaflets). Then each leaflet was visually divided into three-thirds; one-third near the edge of leaflet was considered “tip of leaflet” and the remaining two-thirds were considered “body of leaflet” (Figure 3). Each anatomical region was divided into 4 or 6 sectors to correspond to the leaflets and commissures distribution in BAV and TAV patients, respectively. The total AVC volume was calculated; then AVC distribution was analyzed for each region.

### 2.4. PVL Assessment

Evaluation of PVL severity was performed at the end of the procedure. PVL was considered positive if it was present after completing all interventions. Echocardiographic assessment was performed by a board-certified echocardiographer experienced in TAVI imaging. PVL was classified using color Doppler imaging into trace, mild, moderate, or severe as suggested by the Valve Academic Research Consortium-2 (VARC-2) consensus recommendations [13]. The location of the PVL jets was determined retrospectively by a board-certified echocardiographer and was blinded to the results of AVC volume and distribution analysis.

### 2.5. The Procedure

**Figure 4:**
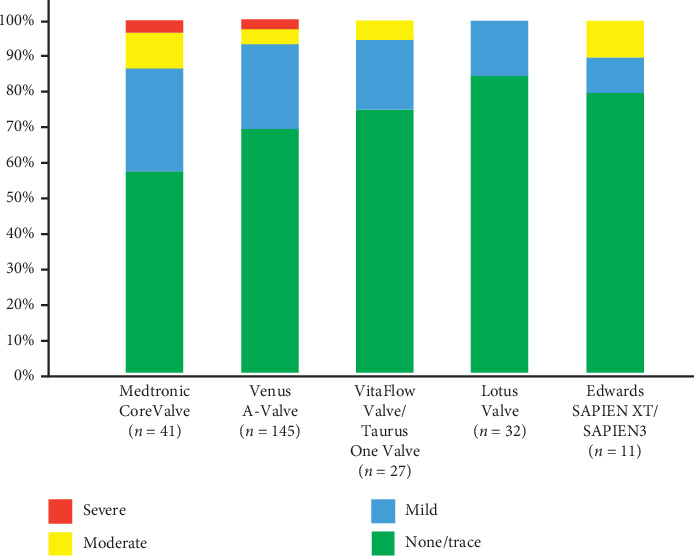
Degree of PVL for each prosthesis type.

Implanted prostheses included the self-expandable prosthesis (the first-generation CoreValve, Medtronic, Inc., Minneapolis, Minnesota. Venus A-Valve, Venus MedTech, Inc., Hangzhou, China. VitaFlow Valve, MicroPort, Inc., Shanghai, China; and Taurus One Valve; Peijia, Inc., Suzhou, China), the mechanical-expandable prosthesis (Lotus Valve; Boston Scientific, Inc., Natick, MA, USA), and Edwards SAPIEN XT or SAPIEN3 valves (Edwards LifeSciences, Inc., Irvine, California, USA) (Figure 4). Prosthesis selection depended on prosthesis availability. Based on the agreement of the heart team, all the patients underwent TAVI using the transfemoral access, except for two patients in whom the transsubclavian and transcarotid approach were used due to unfavorable femoral anatomy. Valve sizing was based on the consensus of a multidisciplinary heart team that includes senior interventional cardiologists, cardiovascular surgeons, and imaging specialists. The need for predilatation was decided by the heart team. Similarly, the choice of postdilatation was at the discretion of the heart team and was generally based on the postdeployment echocardiographic imaging showing significant PVL.

### 2.6. Statistical Analyses

Mean ± standard deviation was used for continuous variables and numbers with percentages for categorical variables. Comparisons between groups were performed with the Chi-square test for categorical variables and Student's *t*-test or Wilcoxon rank-sum test for continuous variables as appropriate. Exploratory multivariable analysis by logistic regression was performed to evaluate the predictors of ≥mild PVL after TAVI. The final model included variables associated with univariate analysis (all variables with a *p*value < 0.1). Statistical analysis was performed using the Statistical Package for Social Sciences, version 21.0, for Windows (SPSS. Chicago, Illinois). All reported *p*values are two-sided and were considered statistically significant if <0.05.

## 3. Results

### 3.1. Baseline Characteristics

Overall, median age was 74 ± 6 years old, and 43.4% were females. The mean STS score was 8 ± 4.35 and NYHA ≥ III in 234 (91.4%) patients. At baseline, the median left ventricular ejection fraction was 54.7 ± 15%, and the mean transvalvular peak velocity was 5 m/s. We observed that those with higher transvalvular peak velocity were associated with PVL after TAVI. The mean pressure gradient dropped from 64 mmHg to 13.7 mmHg immediately after the procedure. Before the procedure, 60 (23.4%) patients had moderate to severe aortic regurgitation, and 43 (16.8%) had moderate to severe mitral regurgitation. Baseline characteristics of patients are shown in Table 1.

### 3.2. Procedural and MSCT Characteristics

Seventy-five patients (29.3%) had ≥mild PVL after the procedure. Of them, 15 patients had moderate PVL and PVL was severe in 2 patients. Among included patients, 213 (83.2%) patients received self-expandable prostheses, 32 (12.5%) received mechanically expandable prostheses, and the remaining 11 (4.3%) patients received balloon-expandable prostheses (Figure 4). By univariate analysis, neither prosthesis type nor size was significantly associated with the occurrence of ≥PVL. MSCT-derived maximum, minimum, mean annular diameters, and annulus area were 27.1 ± 3.14 mm, 21.3 ± 2.75 mm, 24.2 ± 2.63 mm, and 462.7 ± 101.2 mm^2^, respectively. All these annulus parameters were significantly associated with PVL. Interestingly, annular ellipticity, annular area cover index, prosthesis-mismatch index, and LVOT nontubularity were not associated with PVL. The mean total AVC volume was 798 ± 594.5 mm^3^. The overall analysis indicates that AVC volume was strongly associated with PVL (Table 2).

### 3.3. Multivariate Analysis

By multivariate analysis, calcification volume (OR: 1.001 [95% CI: 1.000, 1.002] *p*=0.01) and prosthesis type (self-expandable versus non-self-expandable) (OR: 3.489 [95% CI: 1.096, 11.105] *p*=0.034) were found to be independent predictors of ≥mild PVL after TAVI, although prosthesis type was not associated with PVL by univariate analysis. Multivariate analysis is shown in Table 3.

### 3.4. Calcification Distribution and PVL Jet Location

**Figure 5:**
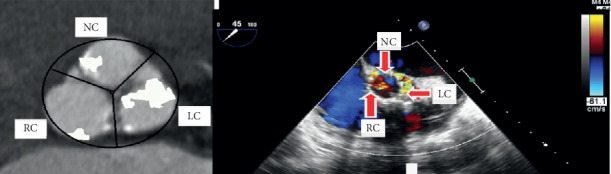
Example showing paravalvular leak jet location on echocardiography matching with the location of AVC on MSCT (image of the MSCT is rotated to be easily compared with the corresponding view of echocardiography). LC: left coronary cusp; RC: right coronary cusp; NC: noncoronary cusp.

**Figure 6:**
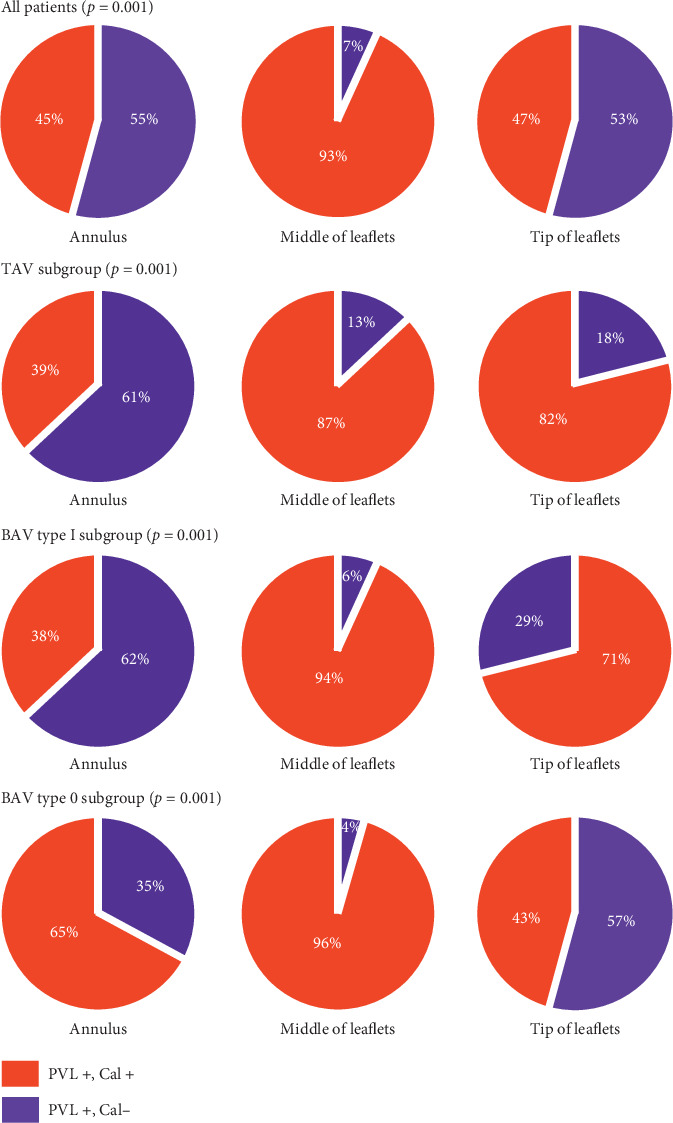
Matching rates of calcification distribution and PVL jet location based on the site of calcification on the aortic root. PVL+, Cal+: paravalvular leak present at the specific location and calcification present at the corresponding location; PVL+, Cal−: paravalvular leak present at a specific location without calcification at the corresponding location; BAV: bicuspid aortic valve; TAV: tricuspid aortic valve.

**Figure 7:**
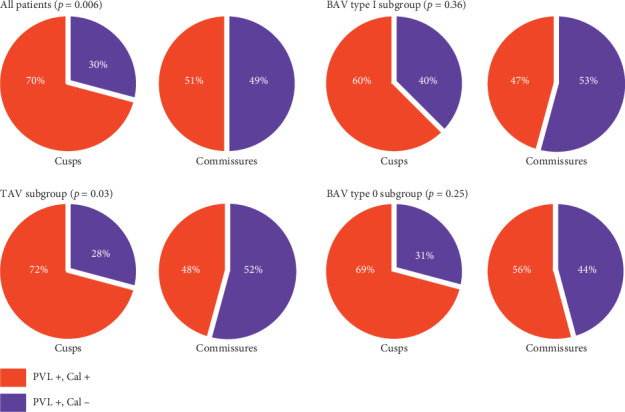
Matching rates of calcification distribution and PVL jet location (Cusps versus commissures). PVL+, Cal+: paravalvular leak present at the specific location and calcification present at the corresponding location; PVL+, Cal−: paravalvular leak present at a specific location without calcification at the corresponding location; BAV: bicuspid aortic valve; TAV: tricuspid aortic valve.

An example illustrating PVL jet location on postprocedural TEE short axes view matching with the location of aortic valve calcification (AVC) on MSCT is shown in Figure 5. PVL jet location matched, in varying proportions, with calcification at all regions of the aortic root, and the highest matching rate was with calcification at body of leaflets compared to calcification at the annulus or tip of leaflets as shown in Figure 6. Matching rates of PVL jet were higher with cusp calcifications than commissure calcifications, particularly in TAV subgroup (Figure 7).

### 3.5. Outcome

At discharge, left ventricular ejection fraction was higher in patients with <mild PVL compared to those with ≥PVL, but no difference was observed in transvalvular valve peak velocity or mean pressure gradient. No statically significant difference in the rate of all-cause and cardiovascular mortality between patients with ≥mild PVL and those with a lesser degree of PVL at 30-day, 6-month, and 1-year follow-up (Table 4).

## 4. Discussion

The main findings of the current study include the following. (1) Risk factors for ≥ mild PVL include AVC volume, larger annulus dimensions, and pre-TAVI transvalvular peak velocity. AVC volume and prosthesis type (self-expandable versus non-self-expandable) were independent predictors of ≥mild PVL. (2) PVL jet matched, in varying proportion, with calcification at all aortic root regions, and the highest matching rate was with calcification at body of leaflets. Moreover, matching rates of PVL jet were higher with cusp calcifications than commissure calcifications, particularly in TAV subgroup. (3) No association between ≥ mild PVL and all-cause and cardiovascular mortality at 1-year follow-up.

### 4.1. Incidence of PVL after TAVI

Despite improvements in TAVI technology, PVL after TAVI remains commonly reported with variable frequencies [3, 14]. This variability was assumed to be due to differences in the imaging modalities used in different centers, evaluation timing, the grading system, and variability in prostheses type [15]. In the current study, 29.3% of patients had ≥mild PVL, which is consistent with several reports [8, 10, 16].

### 4.2. Risk Factors for PVL

Smaller annulus size has been reported to be protective against the presence of PVL, explained by the better congruence between the small annulus and THV. However, the prostheses might be undersized in patients with larger aortic annuli [17]. Results from REVIVAL trial showed that larger aortic annulus was a predictor of post-TAVI central aortic regurgitation rather than PVL due to the requirement of larger postdilatation balloon leading to possible leaflet distortion [18]. Conversely, some publications have reported that larger annulus dimensions were not predictors of PVL [7, 8]. In the present study, larger annulus dimensions were significantly associated with ≥mild PVL. As well as that, a meta-analysis study found that undersizing of the prosthesis relative to the annulus size was the main cause of PVL [3]. However, most of the studies included in their meta-analysis measured aortic annulus using TEE rather than MSCT, which has been proven to underestimate the annulus size [4]. In the present study, however, no statistically significant correlation was found between these parameters and the incidence of PVL, which can be explained by the proper oversizing in our study, as the prosthesis size was always greater than that of the annulus (Table 2). Therefore, these results indicate that an appropriate oversizing based on accurate MSCT-derived annulus measurement is crucial to minimize the incidence of PVL after TAVI. Wong et al. [19] reported that elliptical aortic annulus as a predictor of PVL after TAVI. However, several other studies found no correlation, which is consistent with our results [3, 8].

### 4.3. Calcification Volume and Distribution

The present study evaluated both severity of PVL and PVL location in relation to the distribution of aortic valve calcification. We found that patients with ≥mild PVL had significantly greater calcification in all regions of the aortic valve. Similarly, previous studies have shown that aortic root calcification predicts significant PVL after TAVI [5, 7]. Importantly, several studies suggested that the distribution of calcification on the aortic root is more important than the calcification volume in determining PVL after TAVI [5–7, 20]. However, results of these studies varied; Koos et al. [6] showed that calcium distribution asymmetry had no role in predicting the severity of PVL after TAVI. Ewe et al. [20] found that calcification at the aortic wall near the annulus level was of more importance compared to leaflet calcification in predicting PVL. Marwan et al. [7] reported that annulus calcification was an important determinant in predicting PVL. In addition, they reported no difference in commissure calcification between patients with and without PVL. Khalique et al. [5] used similar methodology like the one we used for classifying the calcification of aortic valve complex and confirmed that both leaflet and annulusLVOT calcification predict significant PVL. The current study found that calcification at all regions of the aortic valve may predict the presence of PVL at the corresponding location. However, calcifications at the body of leaflets were found to be the main determinant in predicting PVL after TAVI. Annulus calcifications and calcifications at the tip of leaflets were less important in predicting PVL. Interestingly, cusp calcifications were found to be more important than commissure calcification in predicting PVL, particularly in TAV and BAV type 0 patients. We believe that leaflet calcification, as suggested by our results and results of a study by Khalique et al. is as important as annular and LVOT calcifications in predicting PVL [5]. The underlying mechanism may be leaflet and annulus/LVOT calcifications causing prosthesis underexpansion and incomplete contact between the prosthesis and its landing zone. In addition, our results suggested that, compared to cusp calcification, commissural calcification is less important in predicting PVL. This finding as suggested by previous report [5] could probably be explained by the fact that contrary to cusp calcifications, commissure calcifications are easier to be pushed outward during the predilatation and deployment procedure and, hence, do not affect the sealing of the prosthesis to its landing zone. We compared the number of patients who underwent predilatation and found that 95% of BAV patients underwent predilatation, in contrast to TAV patients where only 71% (*p* < 0.001) were predilated which may further explain the lower contributing effect of commissure/raphe calcification, in BAV patients, to the development of PVL.

Operators should be cautious when dealing with heavily calcified aortic valves, especially calcifications on areas found to predict PVL after TAVI. In such patients, significant PVL should be anticipated and hopefully prevented by a wise selection of the prosthesis type and proper predilatation to help spread calcified leaflets and preparation for balloon postdilatation and even implantation of a second valve in case of significant PVL.

### 4.4. Prosthesis Type

Widely variable incidence of PVL after TAVI has been observed among patients with both balloon-expandable and self-expandable prostheses. Athappan et al., in their meta-analysis study, found that the incidence of ≥moderate aortic regurgitation after the implantation of self-expandable and balloon-expandable valves was 16% and 9%, respectively [3]. Similarly, a recent study confirmed that aortic regurgitation after TAVI was found to be more frequent in patients with self-expandable prosthesis compared to those with balloon-expandable ones [21]. Conversely, some other studies reported no significant association between prosthesis type and incidence of PVL [8]. In our study, by univariate analysis, prosthesis type had no role in predicting PVL. However, in multivariate analysis, the prosthesis type (self-expandable prosthesis versus non-self-expandable prosthesis) was a predictor of PVL. It should be mentioned that, in the present study, the number of patients who received a self-expandable prosthesis (83%) is significantly greater than those who received another type of prostheses (17%). Hence, it cannot be concluded that a certain prosthesis predicts PVL. Further evaluation of the outcome of different prosthesis types in terms of PVL is warranted using a large and equal number of patients for each prosthesis type.

Yoon et al. found that, in patients with BAV anatomy, new-generation devices were associated with less moderate or severe PVL compared to early-generation devices [22]. Similarly, our results showed that around 40% and 30% of patients who underwent TAVI using CoreValve and Venus A-Valve, respectively, had ≥mild PVL. On the other hand, only less than 25% of those who received new-generation devices had ≥mild PVL after TAVI (Figure 4). Although new-generation devices have less incidence of PVL and, hence, should be preferred over early-generation ones, nevertheless, mild PVL still occur and minimizing PVL is crucial for better outcome of TAVI, particularly in an intermediate-to-low risk patients.

### 4.5. Outcome

There was no difference in terms of all-cause and cardiovascular mortality at 1-year follow-up. This may be explained by the relatively younger age of included patients (mean age was 74 years) and the relatively short follow-up period.

## 5. Conclusions

Risk factors for PVL after TAVI include AVC volume, larger annulus dimensions, and pre-TAVI transvalvular peak velocity. AVC volume is an independent predictor of PVL. Body of leaflet calcifications (versus annulus and tip of leaflet) and cusps calcifications (versus commissures) were more important in predicting PVL. There was no association between ≥mild PVL and 30-day, 6-month, or 1-year all-cause and cardiovascular mortality.

## 6. Limitations

We acknowledge that our study has some limitations. First, this is a retrospective study at one center; we need to be cautious when extrapolating the present findings to other cohorts. Second, most of the included patients underwent TAVI using self-expandable prosthesis. Hence, the study is insufficient to assess the impact of prosthesis type on the incidence of PVL. Third, a relatively short follow-up period makes it hard to estimate mortality, and longer follow-up is warranted. Finally, the number of patients in whom the correlation between calcification distribution and PVL was analyzed was relatively small. This will need to be explored in a larger population.

## Figures and Tables

**Table 1:** Baseline characteristics of patients based on the severity of PVL.

Clinical characteristics		All (*n* = 256)	≥mild PVL (*n* = 75)	<mild PVL (*n* = 181)	*p* value
Age (year)		74 ± 6	73.68 ± 5.89	74.17 ± 6.19	0.56
Female gender		111 (43.4%)	29 (38.7%)	82 (45.3%)	0.33
Body mass index		22.12 ± 3.44	21.88 ± 3.30	22.35 ± 3.59	0.33
STS SCORE		8.01 ± 4.4	8.1 ± 4.01	7.93 ± 4.78	0.80
History of dyspnoea		230 (89.8%)	65 (86.7%)	165 (91.2%)	0.27
History of chest pain		74 (28.9%)	19 (25.3%)	55 (30.4%)	0.42
History of syncope		41 (16.4%)	13 (17.3%)	28 (15.5%)	0.71
Hypertension		114 (44.5%)	37 (49.3%)	77 (42.5%)	0.32
Diabetes mellitus		46 (17.9%)	15 (20%)	31 (17.1%)	0.59
Chronic obstructive pulmonary disease		162 (63.2%)	50 (66.7%)	112 (61.9%)	0.47
Coronary artery disease		110 (42.9%)	32 (42.7%)	78 (43.1%)	0.95
Previous myocardial infarction		5 (1.9%)	2 (2.7%)	3 (1.7%)	0.61
Peripheral vascular disease		143 (55.8%)	35 (46.7%)	108 (59.7%)	0.057
Prior stroke or transient ischemic attack		34 (13.2%)	8 (10.7%)	26 (14.4%)	0.43
Chronic kidney disease		36 (14.0%)	13 (17.3%)	23 (12.7%)	0.33
Atrial fibrillation		37 (14.4%)	9 (12%)	28 (15.5%)	0.47
NYHA				0.88
Class I		1 (0.4%)	0	1 (1.6%)	
Class II		21 (8.2%)	6 (8%)	15 (8.3%)	
Class III		113 (44.1%)	35 (46.7%)	78 (43.1%)	
Class IV		121 (47.2%)	34 (45.3%)	87 (48.1%)	
NYHA III/IV		234 (91.4%)	69 (92%)	165 (91.2%)	0.82
Echocardiographic factors				
Left ventricular ejection fraction (%)		54.7 ± 15	52.93 ± 14.98	56.48 ± 15.12	0.088
Aortic valve peak velocity (m/s)	5 ± 0.73	5.15 ± 0.75	4.94 ± 0.71	0.035
Aortic valve mean pressure gradient (mm Hg)		64.32 ± 19.3	66.56 ± 20.57	62.08 ± 17.97	0.084
Aortic regurgitation (moderate to severe)		60 (23.4%)	24 (26.3%)	36 (19.9%)	0.19
Mitral regurgitation (moderate to severe)		43 (16.8%)	17 (19.5%)	26 (16%)	0.42

Data are presented as mean ± SD or percentages. NYHA: New York Heart Association; STS score: Society of Thoracic Surgeon score.

**Table 2:** Procedural and MSCT characteristics of patients based on the severity of PVL.

Procedural factors	All (*n* = 256)	≥mild PVL (*n* = 75)	<mild PVL (*n* = 181)	*p* value
Annular maximum diameter (mm)	27.1 ± 3.14	27.67 ± 3.19	26.52 ± 3.10	0.01
Annular minimum diameter (mm)	21.3 ± 2.75	21.67 ± 2.71	20.85 ± 2.80	0.03
Annular mean diameter (mm)	24.2 ± 2.63	24.67 ± 2.66	23.69 ± 2.59	0.01
Annular area (mm^2^)	462.7 ± 101.2	481.09 ± 103.24	444.38 ± 99.21	0.001
Annular ellipticity	21.17 ± 8.9	21.33 ± 8.46	21.02 ± 9.47	0.81
Diameter derived prosthesis mismatch (%)	12.8 ± 9.9	12.09 ± 10.46	13.62 ± 9.26	0.25
Prosthesis/mean annulus diameter ratio	1.13 ± 0.10	1.12 ± 0.10	1.14 ± 0.09	0.25
Calcification volume (mm^3^)	798 ± 594.5	991.64 ± 709.94	604.37 ± 479.05	<0.001
Presence of predilatation		150 (82.9%)	66 (88%)	0.30
Area cover index	19.9 ± 14.6	18.66 ± 15.52	21.14 ± 13.66	0.21
Depth of implantation (mm)	6.8 ± 4.43	7.21 ± 4.47	6.40 ± 4.39	0.27
LVOT nontubularity	−5.3 ± 18.46	−7.15 ± 18.59	−3.46 ± 18.33	0.15
Second valve implantation	28 (10.9%)	7 (9.3%)	21 (11.6%)	0.59
Postdilatation	115 (44.9%)	45 (60%)	70 (38.7%)	0.002
Size of the prosthesis (mm)				0.23
23	46 (19.9%)	9 (12.0%)	37 (20.4%)	
26	109 (42.6%)	30 (40%)	79 (43.6%)	
29	74 (28.9%)	26 (34.7%)	48 (26.5%)	
31/32	27 (10.5%)	10 (13.3%)	17 (9.9%)	
Prosthesis type				0.054
Self-expandable	213 (83.2%)	69 (91.9%)	144 (79.6%)	
Mechanically expandable	32 (12.5%)	4 (5.4%)	28 (15.5%)	
Balloon expandable	11 (4.3%)	2 (2.7%)	9 (5.0%)	
Type of native valve				0.61
Tricuspid	114 (44.5%)	31 (41.3%)	83 (45.9%)	
Bicuspid type I	58 (22.6%)	16 (21.3%)	42 (23.2%)	
Bicuspid type 0	84 (32.8%)	28 (37.3%)	56 (30.9%)	

Data are presented as mean ± SD or percentages. LVOT: left ventricular outflow tract.

**Table 3:** Multiple regression analysis.

Variable	OR (95% CI)	*p* value
Peripheral vascular disease	0.644 (0.335–1.237)	0.18
Calcification volume (mm^3^)	1.001 (1.000–1.002)	0.010
Prosthesis type (self-expandable versus non-self-expandable)	3.489 (1.096–11.105)	0.034
Left ventricular ejection fraction (%)	0.981 (0.959–1.004)	0.097
Transvalvular peak velocity (m/s)	1.826 (0.334–9.985)	0.488
Transvalvular mean pressure gradient (mmHg)	0.991 (0.928–1.058)	0.789
Annular maximum diameter (mm)	1.105 (0.835–1.461)	0.486
Annular minimum diameter (mm)	1.118 (0.844–1.483)	0.437
Annular mean diameter (mm)	1.251 (0.712–2.199)	0.437
Annular area (mm^2^)	0.995 (0.982–1.009)	0.50

**Table 4:** Follow-up outcome data.

At discharge	All (*n* = 256)	≥mild PVL (*n* = 75)	<mild PVL (*n* = 181)	*p* value
Transvalvular peak velocity	2.44 ± 1.1	2.39 ± 0.52	2.49 ± 1.59	0.61
Transvalvular mean pressure gradient	13.7 ± 5.9	13.85 ± 5.82	13.49 ± 6.13	0.66
Left ventricular ejection fraction	55.87 ± 12.1	53.67 ± 12.17	58.07 ± 12.04	0.008
30 days				
All-cause morality		5 (6.7%)	5 (2.8%)	0.16
Cardiac-related mortality		4 (5.3%)	3 (1.7%)	0.19
6-months				
All-cause mortality		8 (10.7%)	8 (4.4%)	0.08
Cardiac-related mortality		5 (6.7%)	4 (2.2%)	0.13
1 year				
All-cause mortality		8 (10.7%)	9 (5.0%)	0.11
Cardiac-related mortality		5 (6.7%)	5 (2.8%)	0.16

Data are presented as mean ± SD or percentages.

## Data Availability

The data used to support the findings of this study are available from the corresponding author upon request.
